# Language Entropy Relates to Behavioral and Pupil Indices of Executive Control in Young Adult Bilinguals

**DOI:** 10.3389/fpsyg.2022.864763

**Published:** 2022-05-04

**Authors:** Floor van den Berg, Jelle Brouwer, Thomas B. Tienkamp, Josje Verhagen, Merel Keijzer

**Affiliations:** ^1^Department of Linguistics & English as a Second Language, University of Groningen, Groningen, Netherlands; ^2^Department of Computational Semantics, University of Groningen, Groningen, Netherlands; ^3^Amsterdam Center for Language and Communication, University of Amsterdam, Amsterdam, Netherlands

**Keywords:** bilingualism, executive control, language entropy, individual differences, pupillometry, generalized additive mixed modeling

## Abstract

**Introduction:**

It has been proposed that bilinguals’ language use patterns are differentially associated with executive control. To further examine this, the present study relates the social diversity of bilingual language use to performance on a color-shape switching task (CSST) in a group of bilingual university students with diverse linguistic backgrounds. Crucially, this study used language entropy as a measure of bilinguals’ language use patterns. This continuous measure reflects a spectrum of language use in a variety of social contexts, ranging from compartmentalized use to fully integrated use.

**Methods:**

Language entropy for university and non-university contexts was calculated from questionnaire data on language use. Reaction times (RTs) were measured to calculate global RT and switching and mixing costs on the CSST, representing conflict monitoring, mental set shifting, and goal maintenance, respectively. In addition, this study innovatively recorded a potentially more sensitive measure of set shifting abilities, namely, pupil size during task performance.

**Results:**

Higher university entropy was related to slower global RT. Neither university entropy nor non-university entropy were associated with switching costs as manifested in RTs. However, bilinguals with more compartmentalized language use in non-university contexts showed a larger difference in pupil dilation for switch trials in comparison with non-switch trials. Mixing costs in RTs were reduced for bilinguals with higher diversity of language use in non-university contexts. No such effects were found for university entropy.

**Discussion:**

These results point to the social diversity of bilinguals’ language use as being associated with executive control, but the direction of the effects may depend on social context (university vs. non-university). Importantly, the results also suggest that some of these effects may only be detected by using more sensitive measures, such as pupil dilation. The paper discusses theoretical and practical implications regarding the language entropy measure and the cognitive effects of bilingual experiences more generally, as well as how methodological choices can advance our understanding of these effects.

## Introduction

It has been theorized that the life experience of using more than one language contributes to enhanced domain-general executive control in bilinguals,^1^ as they are constantly required to regulate the simultaneous activation of multiple languages in one mind (Kroll et al., 2012). However, defining “bilingualism” is perhaps an impossible feat (Surrain and Luk, 2019). There is now a general consensus that it is unattainable to accurately represent the dynamic, multifaceted, and complex nature of bilingualism by treating it as a binary construct (Bialystok, 2021, this special issue). Recent work examining bilingualism on a continuum has suggested that individual experiences place different demands on language control and domain-general cognitive systems, each differentially shaping language processing, cognitive functioning, and brain structure and function (DeLuca et al., 2019; Beatty-Martínez and Titone, 2021; Gullifer and Titone, 2021b). Despite recent attempts to unravel the complexity of bilingualism and its consequences for cognition, much remains unknown about how bilingual experiences may be responsible for these neurocognitive adaptations. Importantly, to capture these intricate effects, sensitive methodologies are required (Poarch and Krott, 2019). This study investigates how the social diversity of language use relates to behavioral and pupil indices of executive control in bilinguals.

Bilingual experiences comprise static factors such as age of acquisition (AoA) and number of languages ever learned as well as ongoing, dynamic experiences such as code-switching practices and current language use within and across contexts. These static and dynamic experiences likely interact in modulating cognitive performance in bilinguals, but recent years have seen a particular focus on the diversity of language use, rather than knowledge, in shaping neurocognitive adaptations in bilinguals (Abutalebi and Green, 2016). This idea was put forward by Green and Abutalebi (2013) in the Adaptive Control Hypothesis. Specifically, Green and Abutalebi identified three types of interactional contexts: a single-language context (SLC), a dual-language context (DLC), and a dense code-switching context (DCS). In the SLC, bilinguals use their languages for different purposes and in strictly separate contexts (e.g., communicating in the L1 at home and in the L2 in educational settings). In the DLC, bilinguals engage in highly integrated language contexts in which their languages may be used in a more balanced manner (e.g., speaking both the L1 and L2 at work, but with different people). Finally, in the DCS, language use is also highly integrated, but fewer restrictions are placed on when to use which language and with whom. According to the Adaptive Control Hypothesis, each context places different demands on language- and domain-general executive control in bilinguals, with the DLC being the most challenging for the executive control system.

Empirical work looking at the influence of interactional contexts on executive control has, for instance, found that Spanish-English bilinguals who reside in contexts in which languages are used separately (i.e., an SLC) showed greater reliance on reactive control, whereas bilinguals residing in contexts in which languages are used interchangeably (i.e., both in dual-language and dense code-switching contexts) mostly adopted proactive control strategies (Beatty-Martínez et al., 2020). Similarly, Hartanto and Yang (2016) classified bilinguals into SLC bilinguals and DLC bilinguals and found that DLC bilinguals showed lower switching costs than SLC bilinguals. In a follow-up study, the authors reported that DLC bilingualism predicted enhanced set shifting abilities and that DCS bilinguals were more likely to perform better on tasks requiring inhibitory control and goal maintenance (Hartanto and Yang, 2020). Likewise, Yow and Li (2015) found a relationship between enhanced goal maintenance (operationalized as mixing cost) and more balanced language use in bilinguals. Altogether, these findings suggest that demands that are placed on bilinguals by the environment differentially modulate cognitive adaptations, on an aggregated level and within bilingual groups.

Despite the empirical importance of investigating theoretical propositions in such aggregated groups, individual variation in bilingual language use is perhaps best captured using continuous measures (Luk and Bialystok, 2013). Bilinguals may not always find themselves in a purely SLC or DLC (cf. Lai and O’Brien, 2020), and on an individual level, some social settings may be characterized as DLCs and others as SLCs (e.g., two languages are spoken at home, but only one language is spoken at work). In this light, Gullifer and Titone (2020) proposed a novel continuous measure of the social diversity of language use: language entropy. Entropy is a concept adapted from information theory (Shannon, 1948) and is generally used to quantify the diversity or uncertainty of a phenomenon. Language entropy reflects a spectrum of language use across or between communicative contexts, and it draws on the concepts proposed in the Adaptive Control Hypothesis.^2^ Crucially, language entropy is not restricted to a set number of languages, as its values range from 0 to log *n* (where *n* is the number of languages that entropy is computed over). It is calculated in such a way that it captures the inherent variability in bilingual language use, where the lowest values approximate compartmentalized language use (i.e., only one language is used in a context), and the highest value represents fully integrated language use (i.e., all languages are used equally). In fully compartmentalized contexts, one language is used much more than the other(s) and, as such, the predictability of which language to use is very high. In highly integrated contexts, the languages are used in a more balanced way and so the (appropriate) language to use is less predictable. It then follows that the degree of unpredictability is also affected by the number of languages a person speaks. That is, when all available languages are used in a fully integrated manner, the unpredictability of which language to use increases as the number of available languages increases. The extent to which the management of this unpredictability is needed is argued to drive neurocognitive adaptations, which consequently increase behavioral efficiency and optimize decision making (Gullifer and Titone, 2021a).

However, it is less clear how this continuous measure of the diversity in bilingual language use may be associated executive control. To reiterate, the Adaptive Control Hypothesis posits that, in contexts where the predictability of which language to use is low, bilinguals need to engage domain-general executive control processes to adapt to changing environmental demands (e.g., a change in interlocutor with whom another language needs to be spoken) to a larger extent than in high-predictability contexts. In other words, they must keep speaking the appropriate language without letting their other language(s) interfere (goal maintenance, also termed proactive control), scan the environment for changes (e.g., conflict monitoring), and switch to another language when this is required (mental set shifting, henceforth set shifting). Previously, higher language entropy has been associated with increased reliance on proactive control (Gullifer et al., 2018; Gullifer and Titone, 2021b), and with functional brain patterns related to enhanced conflict monitoring, set shifting, and goal maintenance (Li et al., 2021), underscoring the possible relationship between the diversity of language use and individual differences in executive control. Importantly, language entropy may reflect a distinct aspect of bilingual language use, as it has been shown by Kałamała et al. (2021) that other indices of bilingual language use, such as code-switching and language-mixing habits, only moderately correlated with language entropy.

In the bilingualism literature, conflict monitoring, set shifting, and goal maintenance have been frequently assessed using cued-switching paradigms (Lehtonen et al., 2018), such as the color-shape switching task (CSST). The cued-switching paradigm is difficult enough to result in large RT costs even in young adults (Monsell, 2003). Despite this, reaction times may not always be sensitive enough in capturing individual differences in certain groups. For example, young adults, a commonly studied demographic, showcase less individual variation in cognitive performance and, as such, in RTs, than other age groups (Hultsch et al., 2002). This may be due to the fact that young adults are at their cognitive performance peak (Park et al., 2002; Bialystok et al., 2012). Perhaps unsurprisingly then, behavioral effects of bilingualism have been found least consistently in young adults (Antoniou, 2019). It is therefore paramount that a measurement is used that is sensitive enough to yield relatively large effects and individual variation when studying young adults, while also capturing a form of processing that is expected to be modulated by bilingual experiences.

It is worth mentioning that cognitive effects of bilingualism have been found in brain indices in the absence of behavioral effects between bilingual and monolingual groups, as well as between bilingual groups with different characteristics (e.g., Bialystok, 2017; Lehtonen et al., 2018; DeLuca et al., 2020). Thus, to further increase sensitivity of the assessments, behavioral indices may be supplemented with a proxy of brain activity, such as pupil dilation. Pupil dilation in response to task demands is commonly thought to be modulated by phasic activity in the locus coeruleus-norepinephrine (LC-NE) system (Aston-Jones and Cohen, 2005; van der Wel and van Steenbergen, 2018). The LC-NE system receives information from the orbitofrontal cortex and the anterior cingulate cortex about task demands. In turn, the LC adjusts its activation patterns to ensure that behavioral responses are optimal (Aston-Jones and Cohen, 2005). As such, pupil dilation can serve as a window into processes related to task performance. An increase in pupil size has often been used as an index of higher resource allocation (i.e., increased cognitive effort and attention allocation to complete the task). This effect has been found in a variety of cognitive tasks (for a literature review, see van der Wel and van Steenbergen, 2018). For example, Rondeel et al. (2015) showed that switch trials elicited larger pupil dilation than non-switch trials in a number switch task. To date, there have been no inquiries regarding the cognitive effects of bilingualism on set shifting using pupil dilation as an outcome measure.

The current study’s primary goal is to examine how the social diversity of bilingual language use, as measured by language entropy, relates to executive control in university students with diverse bilingual experiences, using behavioral measures and pupil dilation. The study was conducted in November and December 2020 at the University of Groningen, the Netherlands, when COVID-19 restrictions were in place. Specifically, the data were collected at a time when teaching took place fully online. The University of Groningen’s student population consists mostly of native speakers of Dutch but also includes international students from all over the world (University of Groningen, 2020). This diverse student population is the result of many of the study programs at the University of Groningen being taught exclusively in English. The Dutch student population starts to formally learn English from a young age (the end of primary school or even earlier) and is regularly exposed to the language through media input, as Dutch television subtitles its foreign programs, for instance. At university, students may speak English in the classroom but Dutch or English or yet other languages with their fellow students during breaks. Their multilingual experience may extend to contexts outside of university, as the North of the Netherlands is a highly multilingual region in itself (Schmeets and Cornips, 2021). In this part of the Netherlands, some speak a regional minority language such as Frisian (in the province of Fryslân) or a form of the Low Saxon dialect in addition to Dutch. In sum, the sample that was targeted in this study was linguistically diverse and likely to vary in how they used their languages across social contexts. This allowed us to assess the impact of inter-individual differences in the social diversity of bilingual language use on executive control.

In our study, we used a color-shape switching task (see method below) to measure conflict monitoring, set shifting, and goal maintenance. As described previously, bilinguals who mainly use their languages in separate contexts are not regularly required to monitor the interactional context for linguistic changes. However, bilinguals who use two or more languages within one context need to engage these precise executive control processes more often to appropriately regulate the activation of their languages, thereby possibly increasing their efficiency over time (Green and Abutalebi, 2013). Thus, we predicted bilingual individuals with higher language entropy to demonstrate enhanced conflict monitoring, set shifting, and goal maintenance abilities relative to those individuals whose language use is more compartmentalized. Crucially, the CSST was adapted to allow for simultaneous recording of pupil size over time, permitting an additional, and potentially more sensitive, measure of set shifting in addition to RTs. Behavioral versions of the CSST have been used regularly in this field (see meta-analysis by Lehtonen et al., 2018). However, to our knowledge, only one study has examined set shifting with simultaneous tracking of pupil size (Rondeel et al., 2015). Changes in pupil size occur very slowly and require slower-paced task designs than purely behavioral tasks (Mathôt, 2018; Winn et al., 2018). Therefore, our secondary objective was to validate whether our version of the CSST captured the expected additional effort of completing switch trials over non-switch trials, henceforth denoted as pupil switching cost, and whether a smaller pupil switching cost was related to higher language entropy. In the case of the CSST, we proposed that a smaller difference in pupil size between switch and non-switch trials would reflect enhanced set shifting efficiency. We explored the possibility that increased efficiency in set shifting is visible in the pupil data only, given that pupil size over time may be more sensitive in detecting individual differences than RTs in our young adult sample.

## Materials and Methods

### General Procedure

Fifty-five young adults were recruited for this study at the University of Groningen, the Netherlands, and through posts on a Facebook page targeting research participants in Groningen. Participants enrolled in the study by filling out a short screening questionnaire at home, which simultaneously served to determine their eligibility to participate. Participants were excluded from participation when they reported having (1) reading or learning disorders; (2) uncorrected sight problems (e.g., color blindness); (3) current substance abuse; (4) past traumatic brain injury; and (5) a history of psychological or neurological disorders. Furthermore, participants belonging to a COVID-19 at-risk group (e.g., people with compromised immune systems and/or pulmonary problems) were not eligible to participate, as data were collected during the COVID-19 pandemic (November and December of 2020). Importantly, participants were not selected based on their language background, as the current study aimed to explore the impact of various bilingual experiences on executive control. Hence, our target demographic consisted of students being born in the Netherlands as well as international students. With most degree programs at the University of Groningen teaching (at least partially) in English, no subjects reported exclusive monolingual daily language use; all reported to be bilingual or multilingual and were proficient in English and at least one other language.

Eligible participants first provided written informed consent online. They were then asked to complete an online background questionnaire at home. They were subsequently invited to an experimental laboratory session. In this session, participants completed three eye-tracking tasks, of which the CSST was administered last. Prior to the CSST, participants completed a resting-state measurement and an anti-saccade task (the results of which are not reported here). Task instructions were given in English.

The entire experimental session took approximately 1 h and 45 min to complete, of which 45 min were spent on the CSST. Participants received a monetary compensation of €15 upon session completion and were debriefed on the goals of the study. The study protocol was approved by the Research Ethics Committee (CETO) of the Faculty of Arts at the University of Groningen (reference number: 69895095).

### Participants

Complete data were collected for 44 participants (33 women), aged 18–30 years (*M* = 22.75, SD = 2.78). Demographic variables such as age, gender, educational attainment, and paternal and maternal educational attainment as a proxy of socio-economic status were extracted from the online background questionnaire. Nineteen out of 44 participants reported to have been born in the Netherlands. Sample characteristics, including language background indices, are listed in Table 1.

**Table 1:** Participant demographics and language experience.

	Participants (*n* = 44)			
	*M*	*SD*	min	max
**Demographics**				
Gender	33 female; 11 male			
Age (years)	22.75	2.78	18	30
Educational attainment^1^	3.25	1.40	2	5
Paternal educational attainment^2^	3.89	1.03	1	5
Maternal educational attainment^1^	3.82	1.05	1	5
**Language experience**				
Number of known languages^3^	3.61	1.03	2	5
*Age of Acquisition (AoA)*				
L2 AoA (years)	6.42	3.45	0	19
L3 AoA (*n* = 33; years)	12.30	4.45	0	22
*Proficiency*				
L1 Speaking (1–10)	9.54	0.87	6	10
L2 Speaking (1–10)	7.79	1.97	1	10
L3 Speaking (*n* = 33; 1–10)	4.66	2.65	1	10
*Exposure*				
L1 Exposure (%)	42.32	24.60	5	85
L2 Exposure (%)	43.49	25.88	0	95
L3 Exposure (*n* = 33; %)	10.97	16.11	0	72
*Code-switching habits*	***n* (%)**			
No switching	21 (47.7%)			
Switches on sentence-by-sentence basis	7 (15.9%)			
Switches on word-by-word basis	16 (36.4%)			

1 Scale of 1–6:1 = primary school, 2 = secondary school, 3 = intermediate vocational education/community college, 4 = University of Applied Sciences or equivalent, 5 = university, and 6 = PhD degree.

2 Scale of 1–5: 1 = no secondary school diploma, 2 = secondary school diploma, 3 = some post-secondary education, 4 = post-secondary degree or diploma, or 5 = graduate/PhD degree or professional degree.

3 Participants were able to indicate up to five languages in the language background questionnaire. Therefore, it is possible that they knew more than five languages.

In total, participants reported 14 different first languages (L1s; first language based on reported age of onset of learning). Dutch was most frequent (*n* = 18), followed by English (*n* = 6), Italian, and German (both *n* = 4). The majority (*n* = 32) reported to speak English as their second language (L2). Participants reported speaking English with a generally high proficiency level (scale of 1–10: *M* = 8.42, SD = 1.22, min = 6, max = 10).

There were 10 participants who did not complete the study, either because they did not fill out the background questionnaire (*n* = 1), because of COVID-19 symptoms or COVID-19 restrictions (*n* = 4), technical difficulties (*n* = 2), or a lack of available lab facilities (*n* = 3). Additionally, it was impossible to calculate entropy scores for one participant due to missing data. This last participant’s data were used in the analyses investigating the main effect of trial type in the CSST, however.

### Materials

#### Background Questionnaire

In order to obtain a detailed picture of participants’ language background and usage patterns, a questionnaire was administered online to participants using Qualtrics (Qualtrics, Provo, UT). In addition to questions asking about standard demographic information, the questionnaire included questions from the LEAP-Q 3.0 (Marian et al., 2007) and the Language Social Background Questionnaire (Anderson et al., 2018). This was done to tailor the questionnaire to the University of Groningen context, specifically. For the purposes of the current study, we extracted data pertaining to language use in several contexts (for reading, for speaking, at home, at university, at work, and in social settings), global language exposure, AoA, and self-assessed language proficiency for the L1, L2, and L3. Please see our entry in the Open Science Framework (OSF; see section “Data Availability Statement”) for the complete questionnaire.

#### Color-Shape Switching Task

To tap conflict monitoring, set shifting, and goal maintenance abilities, we used a CSST. In the CSST, participants are presented with colorful geometric figures and are asked to respond to the color (in our case, blue or orange) or the shape (in our case, a circle or a square) of the figure by means of a button-press. In so-called single blocks, participants are required to respond to a single criterion (i.e., only color or only shape). In the color task, participants decide by means of a button-press whether the figure is blue or orange, and in the shape task, participants press a button to indicate whether the figure is a circle or a square. In mixed blocks, a cue indicates to which criterion the participant should respond. These cues randomly alternate within blocks, resulting in switch trials (trials for which the criterion changes) and non-switch trials (trials for which the criterion is the same as for the previous trial).

Following Li et al. (2021), we extracted global RT, switching costs, and mixing costs as indices of executive control. Global RT is represented by the overall RT in the mixed blocks and has been used previously to relate language entropy to conflict monitoring (Li et al., 2021). Switching costs were calculated as the difference in RTs between switch trials and non-switch trials in the mixed blocks and were used as a proxy for set shifting (Prior and MacWhinney, 2010). Mixing costs were calculated by the difference in RTs between non-switch trials in the mixed blocks and single trials and have been considered to tap goal maintenance abilities (Marí-Beffa and Kirkham, 2014). As engaging in contexts where language use is more integrated requires a speaker to monitor the environment for linguistic changes, we expected that bilinguals with more integrated language use would have more efficient conflict monitoring abilities, as manifested in faster global RTs. Furthermore, we predicted that more integrated bilingual language use would be associated with smaller switching and mixing costs in RTs, taking into account the findings by Li et al. (2021), Gullifer et al. (2018), and Gullifer and Titone (2021b).

##### Apparatus

Pupil size over time (in arbitrary units) was recorded using the Eyelink Portable Duo eye-tracking system (SR Research, Canada) at a sampling rate of 500 Hz. Data were only collected for the participants’ dominant eye. The CSST was programmed using OpenSesame version 3.2.8 (Mathôt et al., 2012) and the PyGaze library (Dalmaijer et al., 2014) and was presented on a 17.3-inch laptop with a 1920 × 1080 resolution.

##### Stimuli

In the CSST, participants were presented with blue (RGB: 95, 167, and 252) and orange (RGB: 207, 152, and 24) squares and circles (square: 2.3° × 2.3°; circle: 2.3° diameter), which appeared one-by-one in the middle of the screen on a light gray background (RGB: 155, 155, and 155). Depending on the criterion, the participant had to either decide on the color or the shape of the stimulus by pressing a key. The cues, which only appeared in mixed blocks, were the words “SHAPE” or “COLOR” and appeared in dark gray (RGB: 112, 112, and 112) in Arial (font size: 72) in the middle of the screen.^3^

##### Experimental Procedure

Participants were seated approximately 60 cm from the eye-tracker. Distance to the eye-tracker was tracked online with a target sticker placed on the participant’s forehead. The eye-tracking signal was calibrated and validated using a nine-point procedure before the start of the task. Manual drift correction took place before each experimental block.

Following Prior and MacWhinney (2010), the participants completed two single-task blocks of 36 items each (color and shape), followed by three mixed blocks of 48 trials each, and ended with two single-task blocks of 36 items each. The order of the single-task blocks, as well as the dedicated response keys, were counterbalanced across participants, resulting in four versions of the experiment. Responses were made pressing the “d” and “f” keys with the left hand and the “j” and “k” keys with the right hand. One hand always responded to the “color” criterion and the other always responded to the “shape” criterion. Experimental blocks were preceded by eight practice trials in single-task blocks, and 16 practice trials in mixed blocks. The practice blocks were repeated until the participant reached an accuracy of at least 80%, to ensure a correct understanding of the task. Participants received feedback on their performance during the practice trials only. In total, the experiment contained 144 single-task block trials (72 color and 72 shape task trials) and 144 mixed trials (72 switch and 72 non-switch trials).

**Figure 1:**
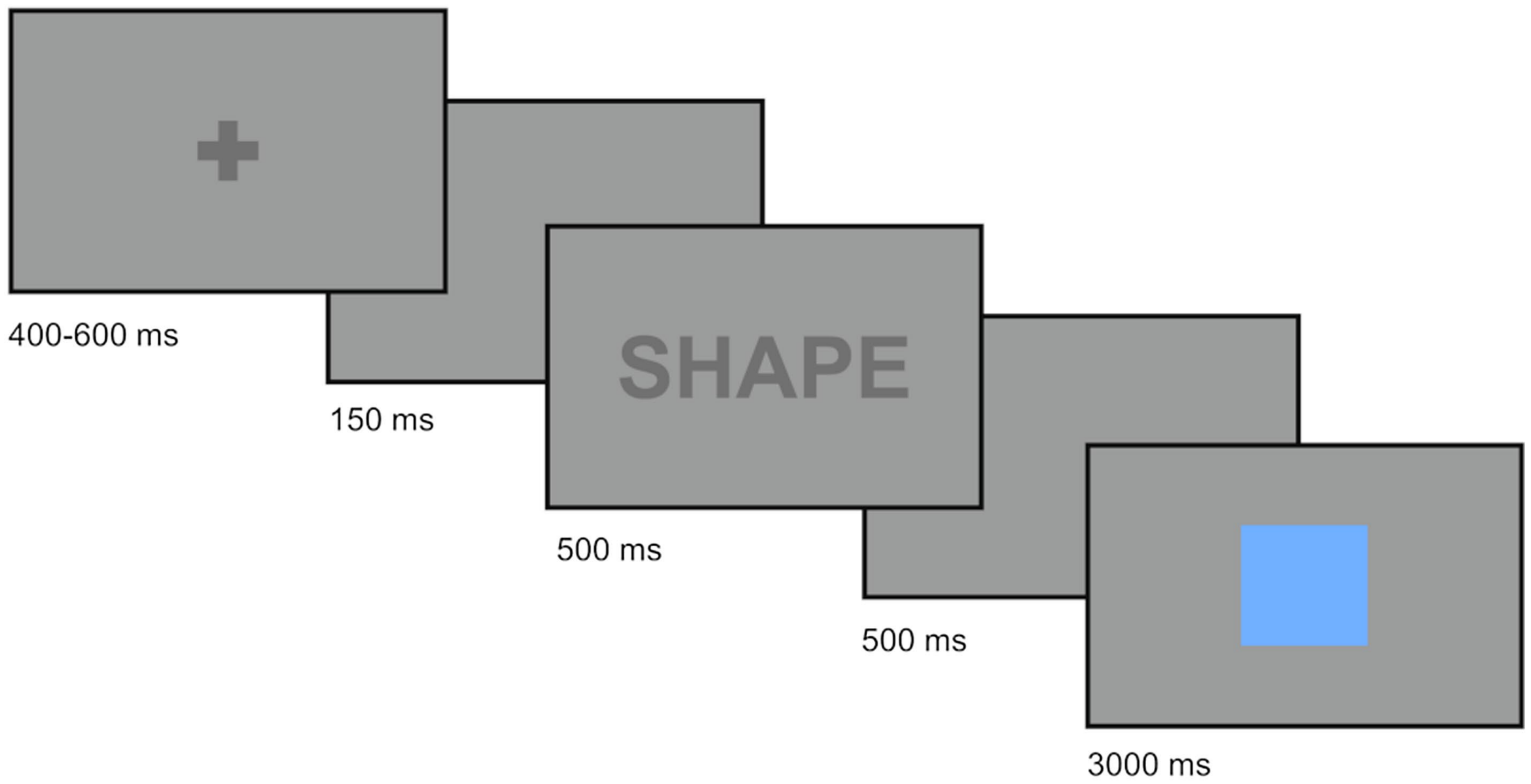
Sample trial procedure for a mixed trial in the color-shape switching task.

Trials were presented as follows. First, the participants looked at a fixation cross at the center of the screen for 400–600 ms in order to trigger the start of the trial. In the single-task blocks, the stimulus appeared after a lag of 150 ms. Alternatively, in mixed blocks, a cue (“COLOR” or “SHAPE”; 500 ms) and an additional gap of 500 ms preceded the stimulus. The stimulus always remained on the screen for 3,000 ms to ensure a fixed trial length within blocks. Despite this, participants were instructed to respond as fast and as accurately as possible. In the mixed blocks, trials of the same type did not appear more than four times in a row. Figure 1 schematically illustrates a mixed block trial.

### Analysis

The data were preprocessed, analyzed, and plotted in R version 4.1.1 (R Core Team, 2021) using version 1.0.7 of the *dplyr* package (Wickham et al., 2018). The full reproducible code is available in the OSF repository.

#### Calculating Language Entropy Scores

Following Gullifer and Titone (2020), language entropy scores were calculated from the self-reported language use data for the L1, L2, and L3 in each communicative context (at home, at university, in social domains, for reading, and for speaking; see Table 2), using the *languageEntropy* package (Gullifer and Titone, 2018). The usage data for the home, university, and social contexts were elicited using Likert scales, with the prompt “Please rate the amount of time you *actively use* the following language(s)/dialect(s) in [context] on a scale of 1–7 (1 = no usage at all, 7 = all the time).” Following Gullifer and Titone (2020), these scores were baselined by subtracting 1 from each response, such that a score of 0 represented “no usage at all.” Subsequently, these scores were converted to proportions by dividing a language’s score by the sum total of the scores in each context. For reading and speaking, language use was elicited by percentage of use (e.g., “When choosing a language/dialect to speak with a person who is equally fluent in all your languages, what percentage of time would you choose to speak each language/dialect?”). All percentages added up to 100%. These percentages were converted to proportions, which were then used to calculate the entropy values per context for each participant. Language entropy was calculated using the entropy formula of Shannon (1948):


$$H=-\overset{n}{\underset{i=1}{\sum }}P_{i}\log_{2}\left(P_{i}\right)$$

**Table 2:** Mean language entropy scores for reading, speaking, home, university, and social contexts.

Language entropy	Participants (*n* = 44)			
	*M*	*SD*	min	max
Reading	0.79	0.38	0	1.57
Speaking	0.74	0.47	0	1.58
Home	0.73	0.47	0	1.58
University	0.43	0.49	0	1.49
Social	0.95	0.37	0	1.58

In this formula, the number of possible languages within the social context is represented by *n*, and *P_i_* is the proportion of the use of language*_i_* in that context. A language entropy value of 0 indicates that only one language is used in a certain context. If a bilingual’s language use is completely balanced, then the entropy value approximates 1 for two languages and 1.60 for three languages.

To reduce the complexity of the entropy data, we followed Gullifer and Titone (2020) and conducted a Principal Component Analysis (PCA). PCA is used to reduce the complexity of a given dataset by grouping correlated variables into a limited set of “principal components” reflecting the variance found in the data set (Abdi and Williams, 2010). We used varimax rotated components and selected our final number of components using a biplot and correlation matrices of the PC scores and individual entropy scores. This resulted in two PC components. Home, social, reading, and speaking entropy loaded into one component and explained 43.2% of the data. University entropy, with some cross-loading from social entropy, loaded into the second component and explained 26.7% of the data. The individual varimax component loadings are provided in the Supplementary Table 1. As a PCA can only be computed over complete cases, work entropy was not included in the PCA, as a considerable number of participants (*n* = 13, 29.5% of the sample) reported to be unemployed. Component scores for each participant were extracted and served as indices of university entropy and entropy anywhere else (non-university entropy) in the subsequent analyses. Recall from above that lower scores represent a more compartmentalized context, whereas higher scores represent a more integrated context, where the proportion of use of each language is more balanced.

#### Preprocessing

##### Behavioral Data

Since participants performed at ceiling level for all trial types (see Section “The Effect of Language Entropy on RTs”), we limited our analyses to RTs. Only RTs from correct responses were analyzed. Following recommendations for RT analysis (Luce, 1991; Whelan, 2008), responses <100 ms were excluded from the analysis (0.38% of the entire dataset). The data were subsetted per trial type to calculate global RT and switching costs (switch and non-switch trials) and mixing costs (non-switch and single-task trials). The processed datasets are available in the OSF repository.

##### Pupil Data

The pupil data collected during the CSST were preprocessed using version 0.0.1.2. of the *gazeR* package (Geller et al., 2020). To preprocess the data, we executed the following steps. First, we identified blinks in the signal and subsequently applied a smoothing function and interpolated the signal using a cubic spline. Then, we applied subtractive baseline correction (pupil size—baseline) for the 200 ms preceding the 150 ms gap in the trial. During the artifact rejection procedure, we excluded 3.98% of the data in the entire dataset in several steps. First, we removed trials that missed >25% of the data. Then, following recommendations by Mathôt et al. (2018), we rejected unlikely pupil values by visually inspecting a histogram of pupil values per participant. Any value that was clearly much higher or lower than the majority of the data was deleted. Lastly, we estimated the mean absolute deviation and removed observations for which the pupil size changed quicker than physiologically probable. As a next step, we aligned the event start time to the presentation of the cue. Finally, we downsampled the data to 50 Hz (i.e., time bins of 20 ms). For a complete discussion and accompanying code of the preprocessing procedure, we refer to our preprocessing script in the OSF repository and Geller et al. (2020).

#### Reaction Times

The RT data were analyzed using a trial-by-trial approach with generalized linear mixed-effects models using the *glmer* function from the *lme4* package (version 1.1-27.1; Bates et al., 2015). *p*-Values of the estimates were obtained *via t*-tests using the Satterthwaite approximations to degrees of freedom, using version 3.1-3 of the *lmerTest* package (Kuznetsova et al., 2017). Following recommendations for RT analysis (Lo and Andrews, 2015), instead of using linear mixed-effects models and log-transforming the RTs, we fitted generalized linear mixed-effects models with an Inverse Gaussian distribution paired with an “identity” link to approximate the distribution of our RT data. We added sum-to-zero orthogonal contrasts to the trial type variable to improve interpretation of the results (Baguley, 2012; Schad et al., 2020). For mixing cost, we coded single trials as −0.5 and non-switch trials as +0.5 (−SI + NS). For switching cost, we coded non-switch trials as −0.5 and switch trials as +0.5 (−NS + SW). As such, the effect of trial type is to be interpreted as the change in effect when moving from one trial type to the other.

To investigate the effect of the diversity of language use at university and in non-university contexts on global RT and switching and mixing costs, we fitted two hypothesis models (RTs for switch and non-switch trials and RTs for non-switch and single trials). RT was entered as the dependent variable, followed by an interaction between trial type (switch and non-switch, or non-switch and single) and university and non-university entropy, a fixed effect of trial number to account for autocorrelation in the data, and a random intercept for each participant. This resulted in the following basic model specification:


RT~(Trial Type×University Entropy)+(Trial Type×Non−university Entropy)+Trial Number+(1|Subject)

Trial number was scaled and centered around the mean in each model. Model comparisons using the *anova* function and the Akaike’s Information Criterion (AIC) assessed whether the addition of random slopes of trial type or trial number per subject improved the fit of each hypothesis model. These random slopes were included in the model to account for the possibility that participants may show individual fatigue effect patterns (i.e., in some participants, RTs may increase as the number of completed trials increases).

Considering that more traditional bilingual language variables may explain variance in the data in addition to language use patterns (Gullifer and Titone, 2020), additional fixed effect predictors of L2 age of acquisition, L2 proficiency, and L2 exposure were added one-by-one to our hypothesis model. These predictors did not significantly contribute to the model fit for switching cost [L2 AoA: (*χ*^2^(1) = 0.2302, *p* = 0.63); L2 proficiency: (*χ*^2^(1) = 0.6773, *p* = 0.41); L2 exposure: (*χ*^2^(1) = 0.1484, *p* = 0.70)] or mixing cost [L2 AoA: (*χ*^2^(1) = 0.8309, *p* = 0.36); L2 proficiency: (*χ*^2^(1) = 0.946, *p* = 0.33)], or inclusion led to unresolvable model convergence issues (in the case of L2 exposure in the mixing cost analysis). Therefore, these predictors were not included in the final models. Model assumptions were checked with version 0.8.0 of the *performance* package (Lüdecke et al., 2021). We applied model criticism on the best fitting models by excluding all observations with absolute residuals larger than 2.5 SDs above the mean (1.99% of the observations for switching cost and 2.14% of the observations for mixing cost). No undue influence from outliers on the model estimates was identified. The final models (see Table 3) reflect the results on the basis of the trimmed datasets. The results were visualized using version 2.8.9 of the *sjPlot* package (Lüdecke, 2021).

**Table 3:** Summary of the *glmer* models of the effect of language entropy on global RT and switching costs (RT) as well as the effect of language entropy on mixing costs (RT) reporting the explained variance and standard deviation (SD) for the random effects, and the model estimates, standard errors (SE), *t*-values, and *p*-values for the fixed effects.

	Global RT and Switching cost				Mixing cost					
**Random effects**										
**Grouping**	**Effect**	**Variance**	**SD**	**Correlation**	**Effect**	**Variance**	**SD**	**Correlation**	
Participant	(Intercept)	9,793	98.960	–	(Intercept)	4,415	66.444	–	
	Trial Type (-NS + SW)	2,413	49.126	0.41	Trial Type (-SI + NS)	4,888	69.912	0.60	
	Trial Number	7,623	87.311	–	Trial Number	348.5	18.668	0.28	0.04
Residual		0.0002903	0.017	–	–	0.0002089	0.0145	–	
**Fixed effects**									
**Effect**	**Estimate**	**SE**	*t*-value	*p*-value	**Estimate**	**SE**	*t*-value	*p*-value	
(Intercept)	851.515	19.937	42.710	**<0.001** ^***^	709.160	12.478	56.834	**<0.001** ^***^	
Trial Type (−NS + SW)	129.358	12.323	10.497	**<0.001** ^***^	–	–	–	–	
Trial Type (−SI + NS)	–	–	–	–	163.158	12.068	13.520	**<0.001** ^***^	
Trial Number	41.743	17.452	2.392	**0.017** ^*^	29.046	5.169	5.619	**<0.001** ^***^	
University Entropy	127.393	27.433	4.644	**<0.001** ^***^	115.336	11.837	9.744	**<0.001** ^***^	
Non-university Entropy	−38.015	27.184	−1.398	0.162	−37.972	11.570	−3.282	**0.001** ^**^	
Trial Type * University Entropy	19.874	19.607	1.014	0.311	23.557	14.206	1.658	0.097	
Trial Type * Non-university Entropy	4.933	19.520	0.253	0.801	−41.526	14.454	−2.873	**0.004** ^**^	

* *p* < 0.05;

** *p* < 0.01;

*** *p* < 0.001. The values in bold reflect significance at at least the *p* < 0.05 level.

#### Pupil Size Over Time

Pupil size over time was analyzed using Generalized Additive Mixed Models (GAMMs).^4^ GAMMs are an extension of mixed-effects regression models (Sóskuthy, 2017). However, they differ in that they are able to model non-linear data using so-called “smooths” (Baayen et al., 2018; Wieling, 2018). These smooths are made by combining a set of basis functions in such a way that they fit the data (for more details, see Wieling, 2018, p. 91). GAMMs then apply a non-linearity penalty to prevent overfitting. This penalty is called wiggliness. This method is especially suitable for analyzing time-course data, as it can take into account autocorrelation and because the signal needs not be averaged over a prespecified epoch. For this reason, GAMMs have become quite popular in recent years for studying event-related potentials (Meulman et al., 2015), dynamic phonetic data (Wieling, 2018), and pupillometric data (van Rij et al., 2019; Boswijk et al., 2020).

GAMMs were fitted in R version 4.1.2 (R Core Team, 2021), using version 1.8-38 of the *mgcv* package (Wood, 2011). First, a base model was built to verify that our version of the CSST captured the additional attentional resources needed to respond to the more difficult switch trials. That is, to see whether switch trials resulted in larger pupil size over time than non-switch trials.^5^ This model included a factor smooth modeling the pupil size over time per participant. Another factor smooth modeled the individual variation over time by trial type. We then investigated if gaze position (i.e., the *x* and *y*-coordinates on the screen), distance to the eye-tracker, and the effect of distance to the eye-tracker per participant needed to be added to the model by comparing AIC scores per model using the *CompareML* function in *mgcv*.

To test our hypotheses, two models were built that included an interaction between trial type with university entropy or non-university entropy. These models were based on the best models resulting from the analysis investigating the main trial type effect. The best fitting models resulting from these comparisons are presented in the Results section. Since the models’ residuals were not normally distributed, all final models were refitted with a scaled-*t* distribution used for heavy-tailed data. The results were visualized using version 2.4 of the *itsadug* package (van Rij et al., 2020). For a complete overview of our model-building procedure, see our entry in the OSF repository.

## Results

### The Effect of Language Entropy on RTs

**Figure 2:**
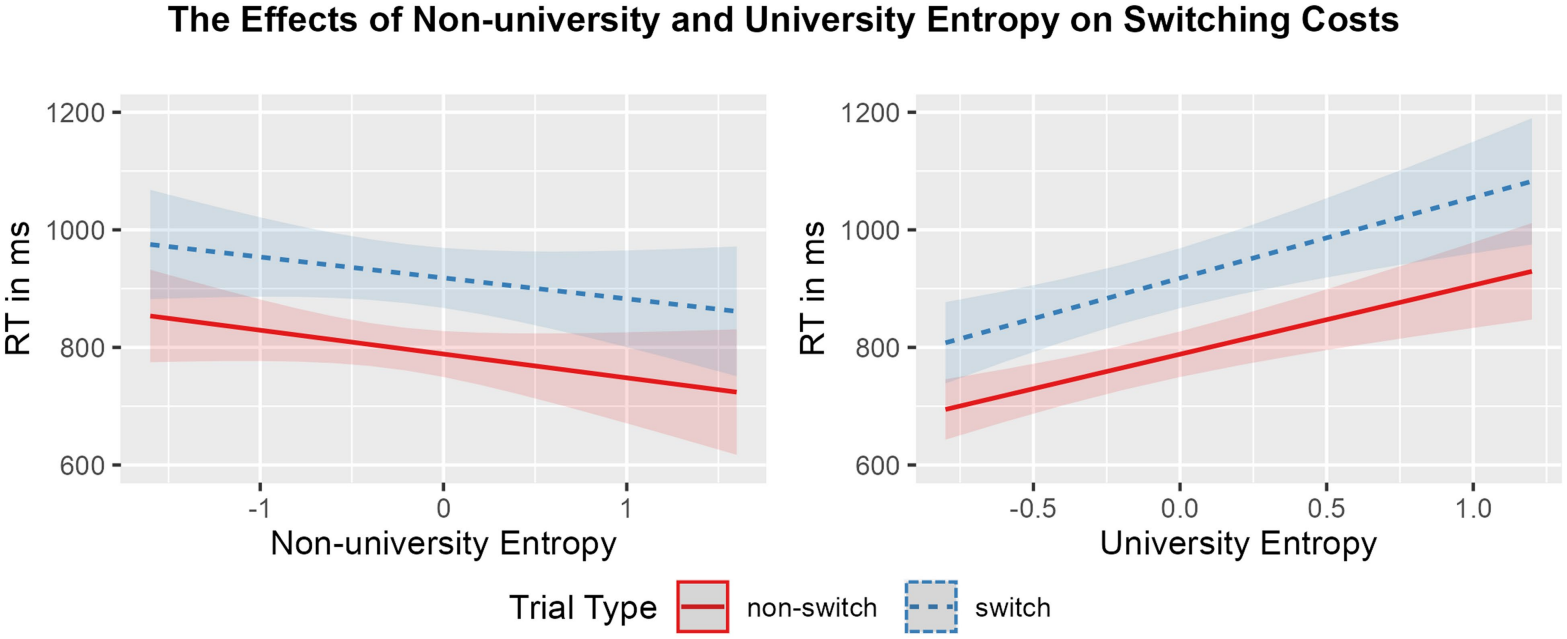
Regression model plot of the interaction between non-university entropy (**left panel**) and university entropy (**right panel**) and trial type (blue striped: switch; red solid: non-switch) on RTs (ms). Shading represents the size of the confidence bands.

**Figure 3:**
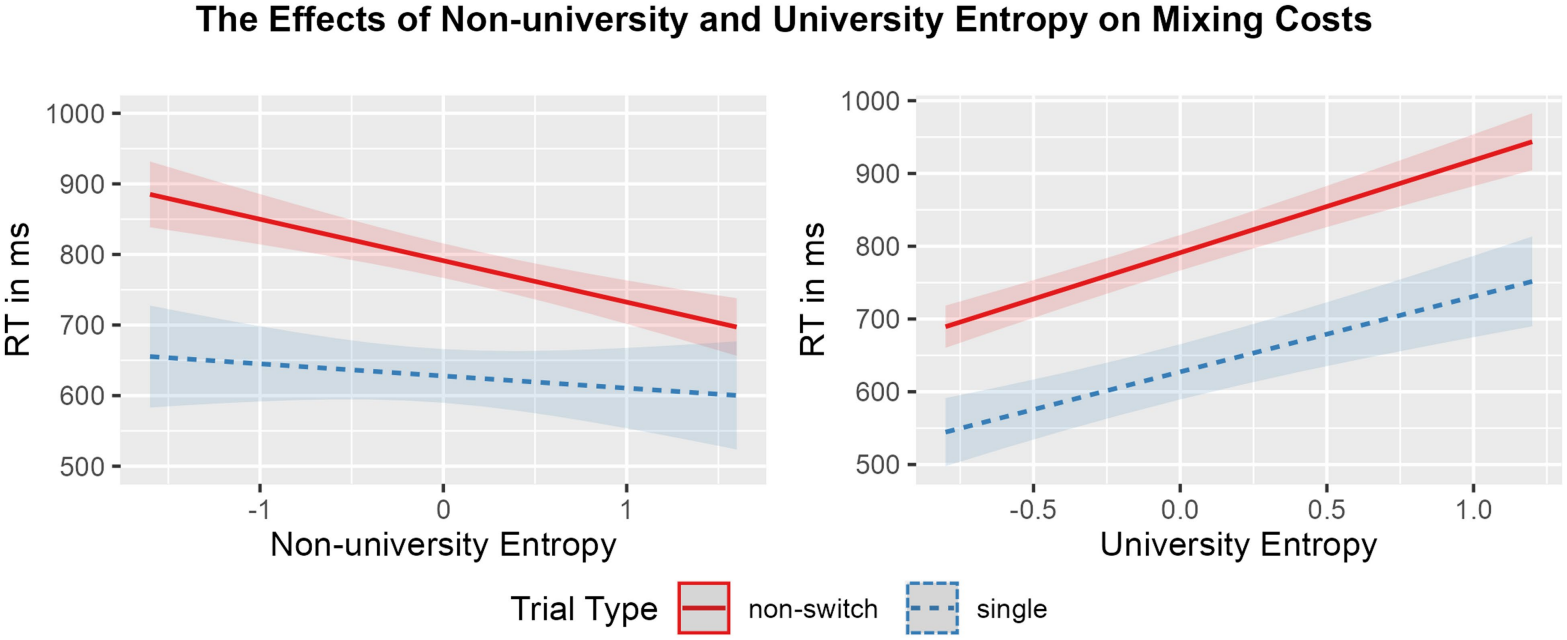
Regression model plot of the interaction between non-university entropy (**left panel**) and university entropy (**right panel**) and trial type (blue striped: single; red solid: non-switch) on RTs (ms). Shading represents the size of the confidence bands.

Mean RTs and accuracy rates per condition, followed by mean global RT, switching costs, and mixing costs in the CSST, are displayed in Table 4. The effects of university entropy and non-university entropy on global RT and switching costs, and on mixing costs are visualized in Figures 2, 3, respectively. Summaries of the final models, including random effects, are available in Table 3.

**Table 4:** Mean RTs (ms) and accuracy, and EF measures derived from the CSST.

	Reaction time (ms) *M* (SD)	Accuracy *M* (SD)
Single-task trials	525.74 (225.23)	0.99 (0.11)
Non-switch trials (mixed block)	624.18 (366.88)	0.97 (0.17)
Switch trials (mixed block)	710.79 (409.77)	0.96 (0.20)
**EF measures**		
Global RT (mixed block)	667.24 (391.17)	
Switching cost	82.92 (505.65)	
Mixing cost	95.57 (426.66)	

The model summary for switching cost showed a main effect of trial type (est = 129.358, *p* < 0.001), such that, overall, participants were slower to respond to switch trials in comparison to non-switch trials (i.e., showed a switching cost, as expected). In addition, university entropy modulated global RT (est = 127.393, *p* < 0.001), suggesting that those individuals with higher diversity of language use at university were generally slower in performing the mixed blocks. No main effect of non-university entropy was found, indicating that non-university entropy did not modulate global RT. Likewise, the interactions between trial type and neither entropy measure were not significant.

Similarly, for mixing cost, a main effect of trial type was found (est = 163.158, *p* < 0.001): participants responded significantly slower to non-switch trials in the mixed block in comparison with single trials (i.e., showed a mixing cost). The results also revealed a main effect of university entropy on RTs (est = 115.336, *p* < 0.001), such that participants who used their languages in a more integrated manner at university were slower in responding overall. The reverse was found for non-university entropy (est = −37.972, *p* < 0.01), indicating that those bilinguals with higher diversity of language use in contexts outside university were faster at responding overall. Finally, non-university entropy interacted with trial type (est = −41.526, *p* < 0.01), such that higher diversity of language use in contexts outside the university setting was related to a smaller mixing cost. No interaction effect was found between university entropy and trial type.

### Pupil Dilation Results

#### The Main Effect of Switching on Pupil Size

**Figure 4:**
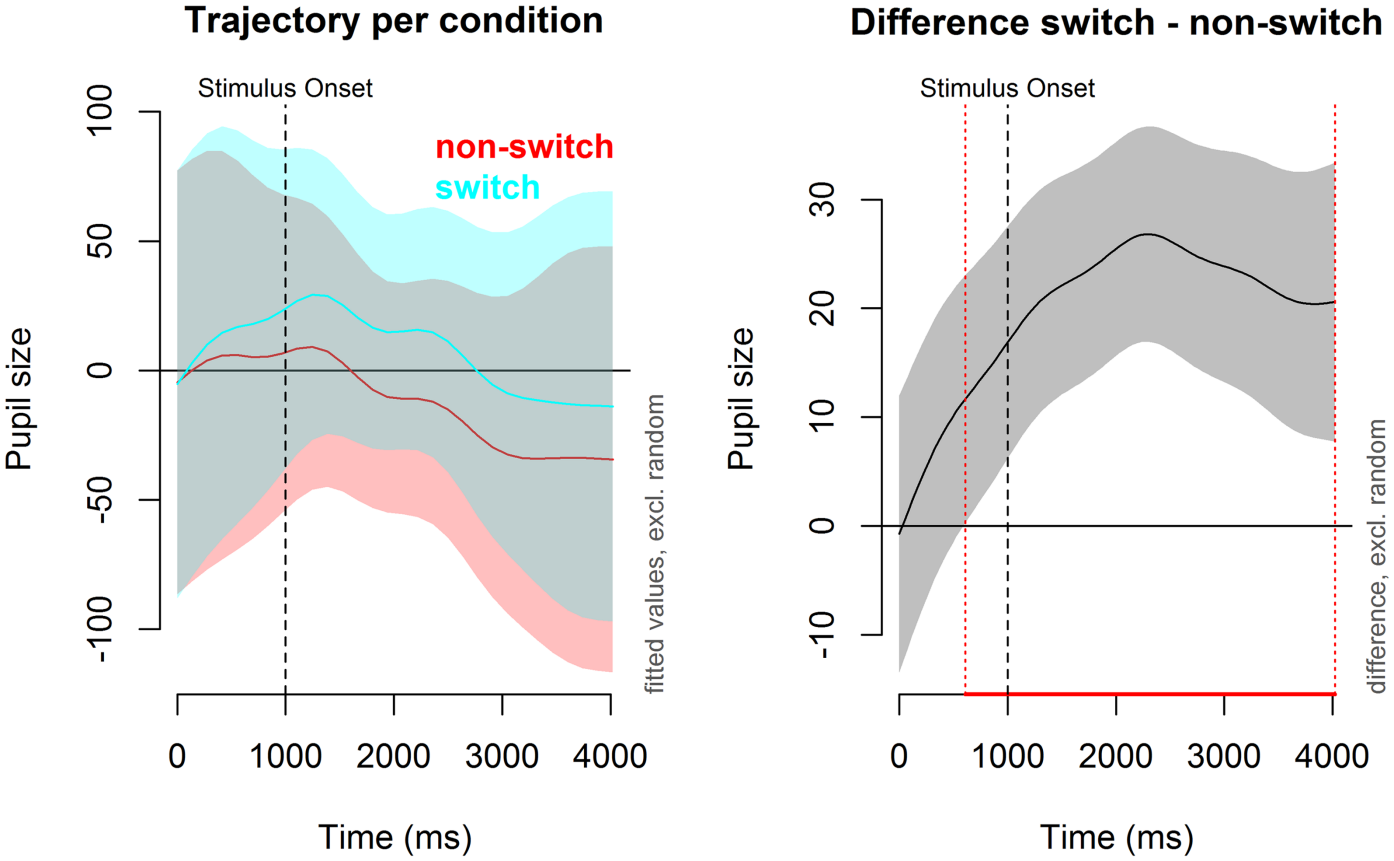
Pupil dilation per trial type over time. **Left panel**: Pupil dilation (in arbitrary units) for switch trials (blue) and non-switch trials (red). Time (*x*-axis) starts at cue onset. The black dotted line at 1,000 ms represents the stimulus onset. **Right panel**: Pupil switching cost. The red dotted line represents the moment the difference in pupil size between switch and non-switch trials became significant.

The first GAMM modeled the main effect of trial type (switch trials versus non-switch trials) on pupil size over time. The results of this model, as well as the interaction models, can be found in Table 5. The average pupil size for switch trials was significantly larger than for non-switch trials (est = 19.591, *p* < 0.001). The model estimates do not tell us how pupil dilation developed over time. In order to evaluate the actual pattern of this non-linear effect during the trial, we plotted the change in pupil size over time for switch trials and non-switch trials in Figure 4. As can be seen in the plots, a pupil switching cost emerged immediately after the cue was shown. The difference between switch and non-switch trials became significant at 609 ms after the cue was shown; it peaked around 2,200 ms, and it remained significant for the remainder of the trial.

**Table 5:** Summary of the generalized additive mixed models looking at the main effect of trial type, and the interaction of trial type with university and non-university entropy on pupil size.

	Base model				University entropy model				Non-university entropy			
	Estimate	SE	*t*-value	Pr(>|*t*|)	Estimate	SE	*t*-value	Pr(>|*t*|)	Estimate	SE	*t*-value	Pr(>|*t*|)
**Parametric coefficients**												
(Intercept)	−7.450	5.859	−1.272	0.204	−10.278	5.261	−1.954	0.051	−7.601	5.214	−1.458	0.145
Trial TypeO—switch	19.591	4.651	4.212	**<0.001** ^***^	–	–	–	–	–	–	–	–
Trial Type—switch	–	–	–	–	12.481	3.435	3.634	**<0.001** ^***^	12.942	3.476	3.724	**<0.001** ^***^
**Smooth terms**	**Edf**	**Ref. df**	*F*-value	*p*-value	**Edf**	**Ref. df**	*F*-value	*p*-value	**Edf**	**Ref. df**	*F*-value	*p*-value
s (Time)	8.959	8.964	28.907	**<0.001** ^***^	–	–	–	–	–	–	–	–
s (Time):Trial TypeO—switch	4.167	4.643	6.673	**<0.001** ^***^	–	–	–	–	–	–	–	–
te (Time, Entropy):Trial Type—non-switch	–	–	–	–	13.394	14.087	0.134	1.00	17.950	18.668	13.241	**<0.001** ^***^
te (Time, Entropy):Trial Type—switch	–	–	–	–	10.996	11.609	1.112	0.349	18.406	19.024	12.381	**<0.001** ^***^
s (Mean X, Mean Y)	28.262	28.960	92.206	**<0.001** ^***^	28.011	28.929	67.604	**<0.001** ^***^	27.979	28.924	63.134	**<0.001** ^***^
s (Mean Distance)	5.486	5.994	3.807	**<0.001** ^***^	5.941	6.465	2.196	**<0.05** ^*^	6.013	6.535	2.539	**<0.05** ^*^
s (Time, Subject)	358.916	395.000	27.324	**<0.001** ^***^	346.851	387.000	28.140	**<0.001** ^***^	344.579	385.000	28.371	**<0.001** ^***^
s (Time, Subject):Trial TypeO—switch	345.950	395.000	4.346	**<0.001** ^***^	316.401	387.000	3.712	**<0.001** ^***^	304.308	385.000	1.620	**<0.001** ^***^
s (Mean Distance, Subject)	165.455	390.000	2.673	**<0.001** ^***^	142.311	383.000	2.064	**<0.001** ^***^	141.529	381.000	2.103	**<0.001** ^***^
	*R*^2^ (adjusted): 0.0939 Explained deviance: 7.22%				*R*^2^ (adjusted): 0.122 Explained deviance: 9.5%				*R*^2^ (adjusted): 0.114 Explained deviance: 8.95%			

We report parametric coefficients, effective degrees of freedom (Edf), reference degrees of freedom (Ref. df), *F*-values, and *p*-values for the tensor products, smooth terms, and random effects. The interaction between university entropy and trial type over time is displayed in two rows. The reason for this is that it can become problematic to have an ordered factor in the fixed effects structure if that same term is also in the model as a non-linear interaction. Also note that the language entropy × trial type interaction in rows 3 and 4 of the smooth terms represent university entropy and non-university entropy, depending on the model.

* *p* < 0.05;

** *p* < 0.01;

*** *p* < 0.001. The values in bold reflect significance at at least the *p* < 0.05 level.

#### University Entropy and Pupil Switching Cost

**Figure 5:**
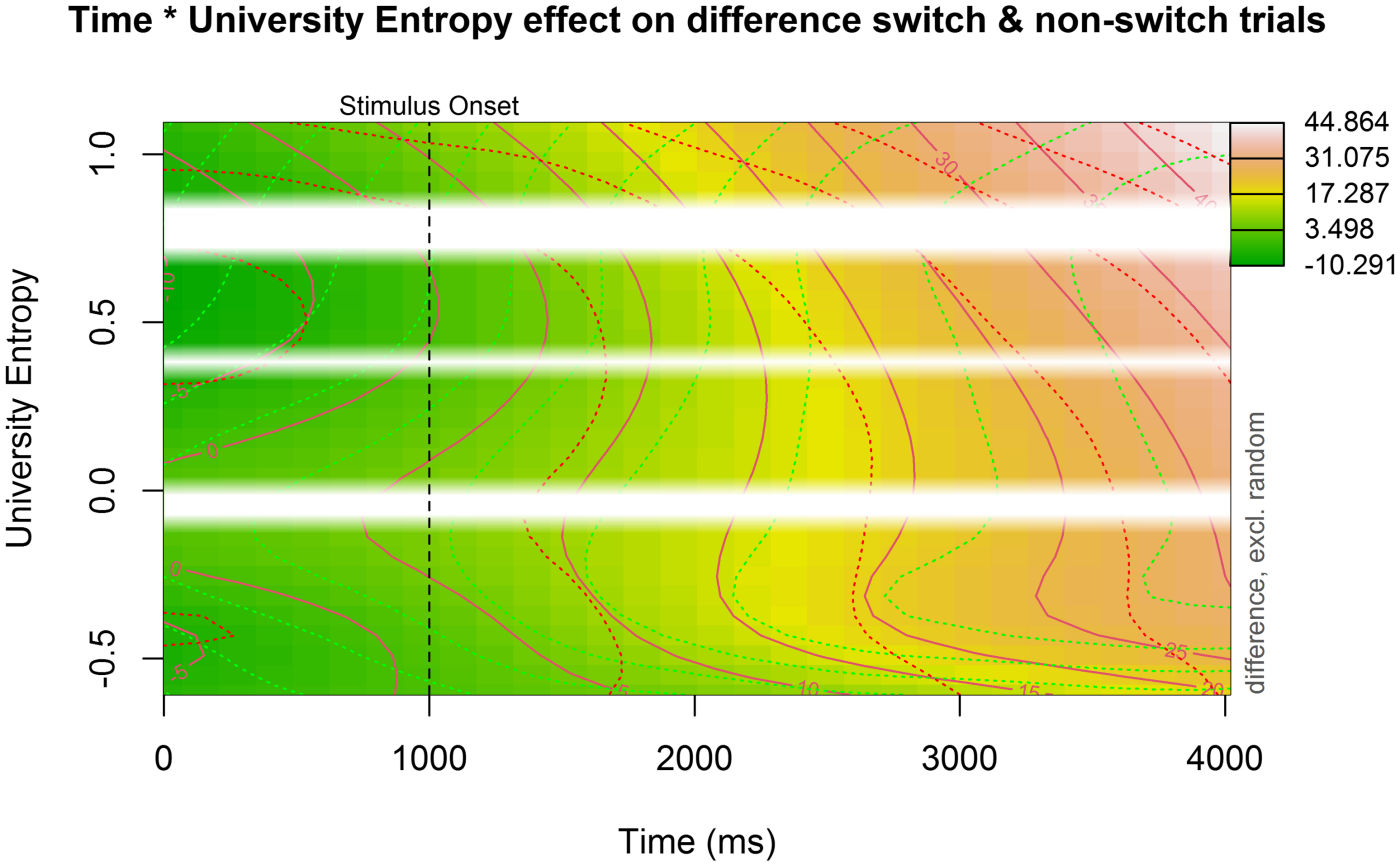
Contour plot showing the interaction between university entropy, time, and the pupil switching cost (i.e., the difference in pupil size between switch and non-switch trials). Time is plotted on the *x*-axis, university entropy is plotted on the *y*-axis, and the pupil switching cost is indicated by color: darker green indicates a small or even reversed effect (where non-switch trials elicit a larger pupil dilation). The more red or even white the plot becomes, the larger the pupil switching cost. The white bars indicate missing data (i.e., non-existing entropy values in our dataset).

The second model supplemented the original model by including a non-linear interaction with university entropy. The model summary can be found in Table 5. The main effect of trial type remained significant (est = 12.481, *p* < 0.001), meaning that the average pupil dilation for switch trials (the reference level) remained larger than for non-switch trials. Figure 5 is a contour plot that models the difference in pupil size between the switch and non-switch trials over time, while taking into account an interaction with university entropy. Contour plots are useful in visualizing three-dimensional interactions, but it is difficult to quantify the size of the difference between switch and non-switch trials based on color alone. The solid lines in the contour plot, therefore, show us how big the difference in pupil size is between switch and non-switch trials. The dotted green and red lines represent the confidence intervals for each line. The pupil switching cost became significant slightly earlier for participants with higher university entropy scores. However, apart from this, there did not appear to be a clear interaction between pupil switching cost and university entropy.

#### Non-university Entropy and Pupil Switching Cost

**Figure 6:**
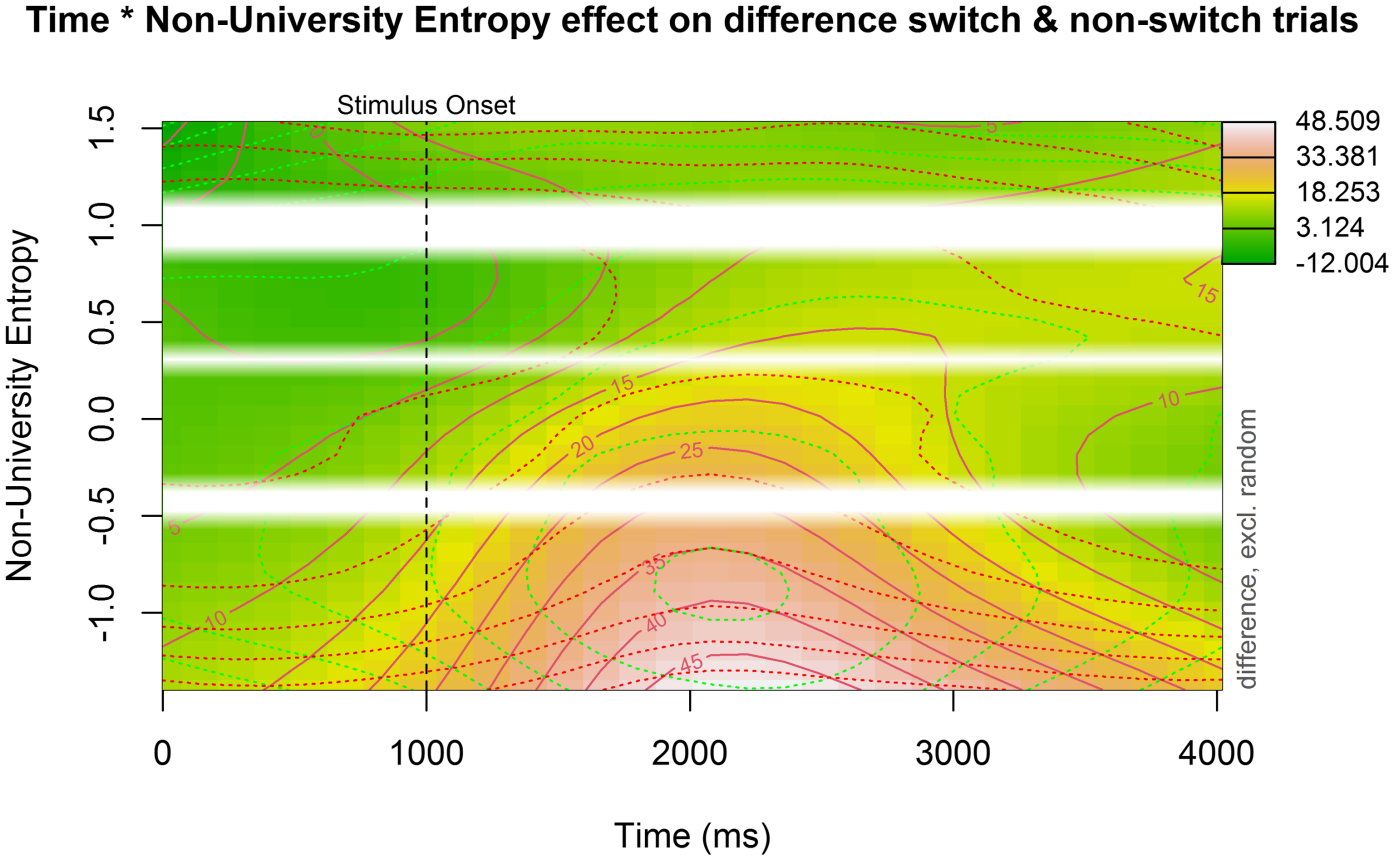
Contour plot showing the interaction between non-university entropy, time, and the pupil switching cost. Time is plotted on the *x*-axis, non-university entropy is plotted on the *y*-axis, and the pupil switching cost is indicated by color: darker green indicates a smaller difference. The more red or even white the plot becomes, the larger the pupil switching cost. The white bars indicate missing data (i.e., non-existing entropy values in our dataset).

The last model supplemented the base model by including a non-linear interaction with non-university entropy. The summary for this model is available in Table 5. The main effect of trial type (est = 12.941, *p* < 0.001) remained, meaning that the average pupil dilation for switch trials continued to be larger than for non-switch trials. To understand the model output, a contour plot was made showing the interaction between non-university entropy and pupil switching cost over time (Figure 6). Participants with lower non-university entropy scores (i.e., more compartmentalized language use) showed a larger pupil switching cost, whereas the difference in pupil size between switch and non-switch trials for participants with higher non-university entropy scores (i.e., more integrated language use) was much smaller. When looking at Supplementary Figure 1, we can deduce that there was no significant difference in pupil size between switch and non-switch trials for participants with the highest non-university entropy scores.

## Discussion

The primary goal of the present study was to examine the effect of the social diversity of language use, as measured by language entropy, on executive control in young adults with diverse bilingual experiences. This was done by administering a CSST, tapping conflict monitoring (global RT), mental set shifting (switching cost), and goal maintenance (mixing cost). We also recorded pupil size over time during the task and compared pupil size during switch and non-switch trials as an additional, and potentially more sensitive measure of set shifting. The social diversity of language use was calculated by looking at self-reported language use in several contexts (at home, speaking, reading, in social settings, and at university). These five contexts were reduced to two components using a PCA, namely, a university entropy component (language use at university) and a non-university entropy component (language use in all other contexts). Based on previous studies, we predicted that language entropy scores would modulate the performance on the CSST, such that individuals who engaged in more integrated language contexts (i.e., had higher entropy scores) would perform the task more efficiently. For RTs, higher university entropy scores were related to slower global RT. In addition, we found reduced mixing costs for individuals with higher non-university entropy scores but not reduced switching costs. However, in the pupillometric data, we found a smaller difference in pupil size between switch trials in comparison with non-switch trials (i.e., a smaller pupil switching cost) for participants with more integrated bilingual language use in non-university contexts. This study is, to the best of our knowledge, the first to provide evidence for the beneficial effects of the diversity of bilingual language use on executive control using pupillometry.

### Language Entropy and Executive Control

Before discussing our primary outcomes, it is important to consider the suitability of the employed method to answer our main research question. In other words, we needed to establish whether the CSST captured robust switching and mixing costs. The pace of the CSST version used in the present study was slower than in previous studies, which was required in order to let the task-induced pupil size return to baseline levels. We therefore took the main effects of trial type on RTs as a starting point for our analysis. The size of the switching and mixing costs was generally smaller than in previous studies using faster paced versions of the task (Prior and MacWhinney, 2010; Hartanto and Yang, 2016; Li et al., 2021). Despite the slower pacing, however, significant switching and mixing costs emerged in our behavioral data. Hence, we assumed that our version of the CSST was able to tap into behavioral indices of conflict monitoring, set shifting, and goal maintenance abilities.

Regarding effects of language entropy on the behavioral measure (RT) of the CSST, our results showed that higher university entropy was associated with slower overall RTs in the mixed blocks (i.e., global RT), contrary to our expectations. These results suggest that those bilinguals with more integrated language use at university showed poorer conflict monitoring skills. Surprisingly, we observed an opposite pattern for the non-university entropy scores, such that bilinguals with higher entropy outside of university were faster at responding in the mixed blocks, albeit not significantly so. These results suggest that the diversity of language use in separate communicative contexts (in our case, university and non-university contexts) may differentially affect executive control. However, we believe there are several potential alternative explanations for these findings. First, our data were collected during the COVID-19 pandemic, when most teaching had been online for several months. Paired with the observation that a number of participants had moved to the Netherlands during the pandemic, it is fair to conclude that these participants only had minimal exposure to university as a social context. Even participants who had been studying at the University of Groningen for more than a year had not taken in-person classes in the 9 months preceding their participation in the current study. Additionally, the quality and quantity of participation in online classes are generally not found to be as high as in-person education (e.g., Meeter et al., 2020). While it is possible that language entropy is not as reliable in assessing bilingual language use in all social contexts, we deem it unlikely that these unexpected results can be attributed to the language entropy measure itself, considering the circumstances. As with any tool, the quality of the measure depends on the quality of the data it is fed. Comparing the university entropy scores to the other examined contexts, we observed a considerable disparity between university and non-university contexts. This was further supported by our PCA that resulted in two clear components with only minimal cross-loading from the other contexts to university entropy. Altogether, this raises the question if the university context was accurately represented as an interactional setting in our study, and consequently, if our outcomes are reliable in this respect.

Regarding the relationship between language entropy and set shifting (as measured by switching cost), there were no significant interactions between either entropy component (university and non-university) and switching cost in the behavioral data. The results are therefore not in line with our prediction that people with higher entropy scores would show a reduced switching cost. These results are not consistent with previous work by Hartanto and Yang (2016) either, who found that DLC bilinguals (i.e., bilinguals with more diverse language use) had significantly lower switching costs than SLC bilinguals. Moreover, our behavioral results do not align with those presented by Li et al. (2021), who found a reduced switching cost for individuals with higher entropy scores. We speculate that the absence of this interaction for switching cost in our study could be caused by the timing of our adapted CSST, as it included a lag of 1,000 ms between the cue onset and stimulus onset to accommodate for the relatively slow pupillary trajectories. Even though our paradigm captured a significant main switching effect, the effect was relatively small (82.9 ms), as compared to 144 ms for bilinguals in Prior and MacWhinney (2010), 199 ms for DLC bilinguals in Hartanto and Yang (2016), and 185 ms in Li et al. (2021). This may be indicative of lower task difficulty, corroborated by the near-ceiling accuracy scores in our task. As such, it could be the case that the relatively small switching effect was not substantial enough to also capture intricate interaction effects, especially if one keeps in mind that there is less individual variation in the RTs of young adults (Hultsch et al., 2002).

Turning to the effects of language entropy on goal maintenance (as measured by mixing cost), no significant interaction was found between university entropy and trial type. Again, we attribute this finding to the possibility that university was not a representative social context during the COVID-19 pandemic. However, we did find a significant interaction between non-university entropy and trial type, such that higher entropy scores were associated with a smaller mixing cost, reflecting enhanced goal maintenance. Interpreted within the Adaptive Control Hypothesis framework (Green and Abutalebi, 2013), our results show that individuals who use their languages in a more integrated manner, and thus encounter situations in which it is less predictable which language will be used, are more efficient in dealing with such ambiguity. These results are in line with our predictions and earlier work demonstrating a relationship between enhanced goal maintenance and more balanced language use (Yow and Li, 2015).

### Language Entropy and Pupil Switching Cost

Our secondary objective was to verify if our version of the CSST, which was adapted for recording pupil size over time, captured the expected additional effort of completing switch trials over non-switch trials, and whether a smaller pupil switching cost was related to higher language entropy. As the CSST had not previously been conducted with pupillometry, the focus of our initial analysis was on the main effect of trial type (switch vs. non-switch trials). As expected, we observed that switch trials induced significantly larger pupillary responses than non-switch trials, thus corroborating the main effect of trial type found in the behavioral data. This suggests that our version of the CSST was able to capture the increased attention that was required for completing the switch trials, and as such, we treated the pupil switching cost as an additional measure of set shifting in our study.

As a next step, we related the language entropy measures to the difference in pupil size for switch and non-switch trials (i.e., pupil switching cost). No interaction effect was found for university entropy and trial type in the pupil data. Several potential reasons for this have been described above. However, the analysis did reveal an interaction effect between non-university entropy and trial type: while a significant pupil switching cost emerged in participants with lower entropy scores, higher entropy scores were associated with smaller, non-significant, and pupil switching costs. This suggests that bilinguals with a higher diversity of language use in non-university contexts showed increased set shifting efficiency. Importantly, this effect was not captured in the RT data. This showcases the benefit of supplementing behavioral data with more sensitive indices, such as pupillometric data, when assessing the cognitive effects of individual bilingual experiences.

The fact that we found a bilingual experience effect that was absent in more traditional behavioral measures is not uncommon in the bilingualism literature (e.g., Bialystok, 2017; Lehtonen et al., 2018; DeLuca et al., 2020). However, it has to be noted that Li et al. (2021) did find a relationship between higher language entropy and a smaller switching cost (but not global RT and mixing cost) in RTs and functional brain patterns relating to executive control. To reiterate, we attribute this discrepancy between earlier work and our study to the faster pacing of the CSST in their study, making it more sensitive in detecting small individual differences in behavioral set shifting than our adapted CSST. Our result also highlights that differences with respect to methodological choices in task design can partly explain mixed results in the bilingualism literature (see Yang et al., 2016, for cued-switching paradigms, specifically).

### Limitations and Future Directions

While the present study presented novel results as to the effects of language entropy on executive control, it was subject to several limitations. First, our study set out to investigate one index of bilingual language use, namely, language entropy. For the current calculation of language entropy, we did not take into account individual differences in the amount of time spent in the communicative contexts. A more accurate picture of the diversity of bilingual language use could be obtained if entropy scores were weighted with the amount of time spent in each social context, as was first done in Kałamała et al. (2020, 2021) and subsequently in Li et al. (2021). This way, one can control for the disparity in engagement in the various contexts. This could be a more appropriate approach, as the diversity of language use in contexts in which an individual spends more time likely has a larger effect on domain-general executive control (Abutalebi and Green, 2016). Moreover, to obtain a more complete image of bilingual language use patterns and their effects on executive control, variables quantifying language switching and mixing behaviors should be considered in conjunction with language entropy (e.g., Kałamała et al., 2021). This would simultaneously enable future research to test the full set of predictions made by the ACH. The second limitation relates to our adapted CSST task. Despite its ability to capture behavioral switching and mixing costs, we propose it can be improved in two ways. First, in its current form, it does not allow for a direct comparison of pupil size during trials in the single blocks and non-switch trials in the mixed blocks to obtain pupil indices of goal maintenance. One way this can be approached in the future is to alter the trial procedure, such that the cue (i.e., “COLOR” or “SHAPE”) is presented in mixed blocks as well as in single blocks. This way, trials are comparable in nature and length across single and mixed blocks, which would enable the investigation of a “pupil mixing cost.” Second, the relatively long lag between cue onset and stimulus onset may explain the lack of an interaction effect between entropy and switching cost in the RT data in our study. This lag was initially introduced to accommodate for expected slow changes in pupil size. However, in the pupillometry analyses, we found that larger pupil dilation for switch trials occurred almost immediately after cue onset, and even that this difference became significant before the stimulus onset. This result strongly suggests that pupillometry is an appropriate way to measure an increase in effort exerted during switch trials. However, it is also likely that the slower pace of our CSST made the task easier to complete, which would explain the generally smaller switching and mixing costs in the RT data, as compared to other studies (Prior and MacWhinney, 2010; Hartanto and Yang, 2016; Li et al., 2021). To capture the behavioral effects better while still measuring pupil dilation patterns over time, we recommend a faster paced design in future studies. Such a design will shorten the task and also increase task demands, possibly leading to an optimal sensitivity in capturing behavioral and pupil size effects.

A final and obvious limitation to discuss is the fact that this study was conducted during the COVID-19 pandemic. Our results currently point toward the possibility that the diversity of language use in separate social contexts (university and non-university contexts) is differentially associated with executive control. This suggests that language use varying per social context may be a key variable in neurocognitive adaptations resulting from bilingual experiences. It could be argued that there is a difference between the two components in terms of voluntarity of language use. While language use in non-university contexts may be more of a choice, students at the University of Groningen are often required to speak English during class, and so it is more predictable when which language to use at university than in other contexts. However, as discussed above, we question the validity of the university entropy component in our study due to the circumstances imposed on the university system during the pandemic. It is therefore difficult to speculate if the contradictory results can indeed be explained as such. Hence, we recommend that future work replicates this study when restrictions regarding in-person teaching have been lifted.

## Conclusion

In conclusion, our study’s findings provide further evidence for the relationship between the social diversity of bilingual language use, as measured by language entropy, and executive control. We demonstrated reduced switching and mixing costs, reflecting enhanced set shifting and goal maintenance abilities, for bilinguals with a higher diversity of language use relative to lower diversity in non-university contexts. No such relationship was found for university contexts, but higher university entropy was associated with weaker conflict monitoring. This potentially illustrates that the effect of the diversity of language use differs per social context. Alternatively, it is possible that university simply was not a valid social context during the COVID-19 pandemic. As such, replication of this study is warranted. We also showed that our adapted CSST effectively captured switching and mixing cost in the RTs. The pupillometry data were able to capture effects that were not visible in the behavioral data. These findings additionally highlight the utility of pupillometry as a sufficiently sensitive tool to assess the effects of individual bilingual experiences on executive control.

## Data Availability Statement

The datasets presented in this study can be found in online repositories. The names of the repository/repositories and accession number(s) can be found at: Open Science Framework repository, https://osf.io/3xjsq/?view_only=e88ee7956d4b4a038bba3cbe59aa871c.

## Ethics Statement

The studies involving human participants were reviewed and approved by the Research Ethics Committee (CETO) of the Faculty of Arts of the University of Groningen. The participants provided their written informed consent to participate in this study.

## Author Contributions

FvdB, JB, and MK conceived the study idea. FvdB and JB developed the methodology. FvdB programmed the experiments. FvdB, JB, and TT carried out the experiments, analyzed the data, and drafted the manuscript. All authors discussed and interpreted the results of the study, critically revised the article, and have read and approved the final manuscript.

## Funding

This research was supported by the Dutch Research Council (grant number: NWO 016.Vidi.185.190) awarded to MK, and by the Center for Language and Cognition at the University of Groningen. The funders had no active role in the design, execution, or analysis of the research.

## Conflict of Interest

The authors declare that the research was conducted in the absence of any commercial or financial relationships that could be construed as a potential conflict of interest.

## Publisher’s Note

All claims expressed in this article are solely those of the authors and do not necessarily represent those of their affiliated organizations, or those of the publisher, the editors and the reviewers. Any product that may be evaluated in this article, or claim that may be made by its manufacturer, is not guaranteed or endorsed by the publisher.

## Footnotes

^1^Our paper uses the term “bilingualism” to represent the proficiency in more than one language, whether the proficiency is in two languages (bilingualism) or in three or more languages (multilingualism).

^2^It needs to be noted, however, that there is no one-to-one mapping of language entropy and the interactional contexts posited in the Adaptive Control Hypothesis, as language entropy does not differentiate between DLC and DCS. For example, language entropy is not able to distinguish a person frequently switching between two languages with one person in one context from a person perfectly balancing speaking two languages in one context with two different people. The resulting entropy values would be comparable.

^3^Whereas in previous versions of the CSST, graphic cues are used to circumvent possible linguistic effects (Yang et al., 2016); for the purpose of our experiment, it was vital to keep the luminosity of the cues constant across conditions. As such, we opted for words denoting the task (cf. Ramos et al., 2017).

^4^For introductions and tutorials for GAMMs, please refer to Sóskuthy (2017), Wood (2017), and Wieling (2018).

^5^The current design of the CSST did not permit appropriate comparison of pupil size during single and non-switch trials. As such, we only target the difference in pupil size for switch- and non-switch trials.
