# Supplementary material for: Language Entropy Relates to Behavioral and Pupil Indices of Executive Control in Young Adult Bilinguals
Source: Front Psychol. 2022 May 4;13:864763. doi: 10.3389/fpsyg.2022.864763 (PMC9116486; doi:10.3389/fpsyg.2022.864763)
Supplement: Supplementary file 1 [file Data_Sheet_1.pdf]

Supplementary Materials

Supplementary Table 1. Varimax rotated component loadings resulting from the PCA.

	**Principal Component**	
**Entropy component**	Non-university	University
Home	0.62031745	0.03905842
University	0.08591523	0.96095558
Social	0.40314488	0.03931195
Speaking	0.57936122	-0.25844937
Reading	0.33111874	0.08183710

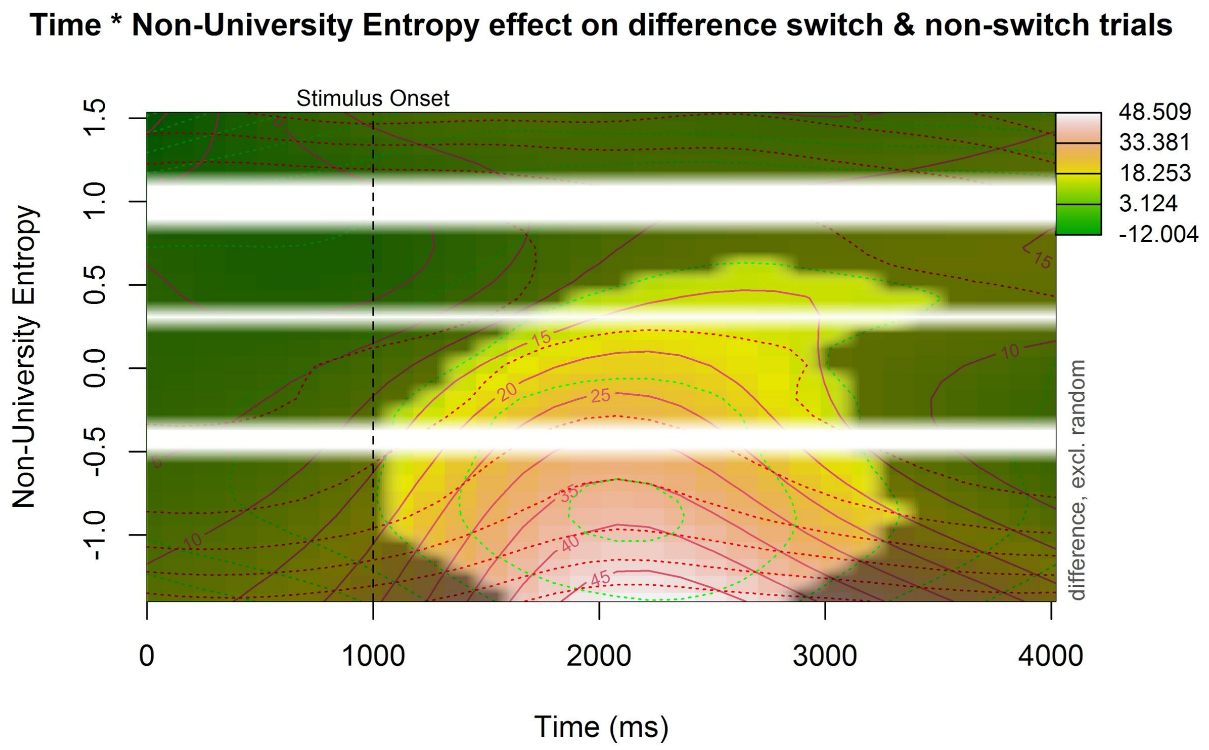

Supplementary Figure 1. Contour plot showing the interaction between non-university entropy, time, and the pupil switching cost. Time is plotted on the x-axis, university entropy is plotted on the y-axis, and the pupil switching cost is indicated by color: the more red or even white the plot becomes, the larger the pupil switching cost. The highlighted area indicates where the difference in pupil size is significant. The white bars indicate missing data (i.e., non-existing entropy values).
